# Functional alignment achieves a more balanced total knee arthroplasty than either mechanical alignment or kinematic alignment prior to soft tissue releases

**DOI:** 10.1007/s00167-022-07156-3

**Published:** 2022-09-18

**Authors:** Gavin Clark, Richard Steer, David Wood

**Affiliations:** 1 St John of God Hospital Subiaco and Midland Hospitals, Subiaco, WA Australia; 2 University of Western Australia, 35 Stirling Hwy, Crawley, WA Australia; 3 Perth Hip and Knee Clinic, 1 Wexford St, Subiaco, WA Australia; 4 Gold Coast University Hospital, 1 Hospital Boulevard, Southport, QLD Australia; 5 University of Queensland, St Lucia, QLD Australia

**Keywords:** Functional alignment, Kinematic alignment, Mechanical alignment, Total knee arthroplasty, Robotic assisted total knee arthroplasty, Balance

## Abstract

**Purpose:**

Total knee arthroplasty with functional alignment uses pre-resection balancing to determine component position within the soft tissue envelope to achieve balance and restoration of native joint obliquity. The purpose of this study was to assess the balance achievable with a mechanical axis alignment and kinematic axis alignment plan, and the subsequent balance achievable after adjustment of the component position to functional alignment.

**Methods:**

A prospective cohort of 300 knees undergoing cruciate retaining total knee arthroplasty were included in this study. Of these, 130 were initially planned with mechanical alignment (MA) and 170 with kinematic alignment (KA). Maximal stressed virtual gaps were collected using an optical tracking software system. The gaps were measured medially and laterally in flexion and extension. Following assessment of balance, implant position was adjusted to balance the soft tissues in functional alignment (FA) and the maximal gaps reassessed. Gaps were considered to be balanced when within 2 mm of equality. Incidence of balance within each cohort was compared to independent samples proportions test.

**Results:**

Functional alignment obtained significantly better balance in extension, medially and overall than both MA and KA alignment without soft tissue release (*p* < 0.001). Overall balance was observed in 97% of FA knees, 73% of KA knees and in 55% of MA knees. The difference between KA and MA was also significant (*p* = 0.002). Whilst there was no difference observed in balanced achieved or limb alignment when FA was planned with either MA or KA, the joint line obliquity was maintained with an initial KA plan.

**Conclusion:**

Functional alignment more consistently achieves a balanced total knee arthroplasty than either mechanical alignment or kinematic alignment prior to undertaking soft tissue release. Utilising an individualised KA plan allows FA to best achieve the stated goals of maintaining joint line plane and obliquity.

**Level of evidence:**

Level III: retrospective cohort study.

## Introduction

As surgeons compare techniques of alignment to optimise total knee arthroplasty (TKA) outcomes it is important to revisit the basic principles. The concept of a balanced knee has long been held fundamental to successful TKA [24]. This has been achieved using a variety of techniques, but the goal has remained unchanged.

To this point, the evidence for balanced extension gaps remains strong with unbalanced knees having poorer gait, patient reported outcome measures (PROMs) and range of motion (ROM) [9, 20]. A surgeon’s ability to assess a balanced TKA is poor [7, 16], even by experienced surgeons [18]. A balanced knee has previously been defined as a gap differential of ≤ 2 mm [8].

Mechanical axis (MA) alignment had the aim of preventing failure through aseptic loosening and fracture associated with tibial varus alignment. This technique altered joint line obliquity and component rotation to bring the joint line perpendicular to the mechanical axis. This technique does not replicate native anatomy with respect to coronal alignment or joint line obliquity (JLO) [17].

Although historically, most discussion has centred around coronal limb alignment in TKA, more recently increasing analysis is centred around the native JLO [11, 17]. This is a combination of the native medial proximal tibial angle (MTPA) and the lateral distal femoral angle (LDFA). Consideration of an individual’s original joint line in TKA has been an influence on various individualised alignment philosophies including Kinematic Axis (KA) [12] and Functional Alignment (FA) [22].

Kinematic axis (KA) alignment has been increasingly adopted as an alternative to mechanical axis alignment [1]. This aims to replicate the bony anatomy of the knee with equal resections of bone distally and posteriorly from both femoral condyles as well as equal resections medially and laterally for the tibial cut. Balance is then assessed, and adjustments to the tibial, but not the femoral cuts can be made to improve balance [13]. Any further balancing is done with soft tissue release.

Functional alignment (FA) has recently been described in the literature as an alternative alignment technique. FA aims to restore the plane and obliquity of the native joint line and it places the balance of the soft tissues as the primary goal of alignment [4, 22]. This technique requires the ability to assess soft tissue balance prior to bone resection, then adjust component position accordingly and accurately execute the surgical plan. The optimal technique for achieving FA is yet to be determined with variation in the literature, utilising either a MA or KA initial plan from which to adjust component position for balance [3, 4].

Currently the literature on FA fails to demonstrate the extent to which FA optimises balance prior to soft tissue balance. Also, how the starting plan influences component position and final balance in FA has not been described. The remaining deficit in current evidence is whether the pre-resection planned balance can be maintained after execution of the TKA.

This study firstly seeks to test the hypothesis that FA TKA are significantly more balanced than either MA or KA TKA prior to soft tissue releases. As secondary outcomes, the study will look to determine whether there is a difference in final implant alignment between FA TKAs balanced from an MA plan [FA(m)] compared to FA TKAs balanced from a KA plan [FA(k)], and whether balance determined on the initial virtual model is maintained to the completion of the procedure.

## Methods

Ethics approval has been granted by the hospital group HREC (SJGHC HREC ref1388). Data management and patient privacy considerations have been adhered to. Participants have consented to prospective data collection and subsequent analysis.

300 consecutive patients with osteoarthritis undergoing robotic assisted primary TKA by a single senior arthroplasty surgeon from January 2017 until August 2018, who were assessed as requiring TKA having failed non-operative management were included. Two patients with previous high tibial osteotomies were excluded. There were no exclusions based on age, body mass index or other patient demographics. This was a sequential cohort with the first group of 130 patients planned with MA alignment and the remainder planned with KA alignment. The change in planning was due to a change in surgeon preferences.

A pre-operative CT scan is used to define the hip and ankle centres along with developing a three-dimensional model of the knee. Pre-operative planning of the total knee arthroplasty is undertaken with Mako software (Stryker, Fort Lauderdale, FL, USA) to plan the bone resections for the preferred implant positioning. Alignment, resection depths and sizing of implants can all be adjusted to optimise the preferences of the surgeon.

MA knees were planned with bone resections of 8 mm from the most distal and posterior points of the femoral condyles and neutral valgus, parallel to the surgical trans-epicondylar axis (SEA), femoral flexion between 0 and 7 adjusted to optimise for best sizing of the femoral component. A tibial resection of 7 mm off the higher side with neutral coronal alignment, 3 posterior slope and rotation set to Akagi’s line.

KA knees were planned with bone resections of 6.5 mm equally from the most distal and posterior points of the femoral condyles. Intra-operatively this was adjusted for bone loss, based on the manual calliper technique described by Howell [13]. Femoral flexion was altered between 0 and 7 adjusted to optimise sizing of the femoral component. Tibial resection was set to 7 mm equal resections medially and laterally, again adjusted for bone loss, with slope set between 0 and 7 based on the native lateral tibial plateau, and rotation set to Akagi’s line [2].

Trans-osseus pins are placed into the tibia and femur, and arrays attached for navigation. A medial parapatellar approach was used in all cases, with minimal medial soft tissue dissection, and ACL resected prior to balancing. Bony landmarks are registered and validated against the patient’s 3D CT model in the planning software (Stryker Mako, Fort Lauderdale, FL, USA). Osteophytes are then removed.

Soft tissue balance of the knee is then assessed with the planned component position by placing varus and valgus stress on the knee to obtain maximal gaps; medially and laterally in extension (approximately 10 degrees, to de-tension the posterior capsule), then medially and laterally in flexion (90°). The software measures the virtual gaps between bone cuts (to nearest mm).

Following assessment of balance with either MA or KA initial plan, the component positioning is adjusted to FA by first balancing extension gaps with coronal adjustment of tibia until balanced or an alignment boundary reached (see Table 1). If further adjustment is required, the coronal alignment of the femoral component was then altered. If balance was not achieved within these boundaries the less diseased side was set to the target gap (20 mm) with the more diseased side left tighter than desired allowing for soft tissue release at the trialling phase.

**Table 1:** Final component alignment boundaries (varus negative, valgus positive)

Coronal limits	
Hip knee ankle angle	− 6° to + 3°
Femoral component	+ 6° to − 3°
Tibial component	− 6° to + 3°
Sagittal limits	
Femoral flexion	0–7°
Tibial slope (KA plans only)	0–7°
Combined component flexion	10°
Femoral rotation (from surgical epicondylar axis)	− 6° to + 6°

Once extension is balanced, medial flexion gap was balanced to be equal to extension gaps. The lateral flexion gap was left anatomical due to the large natural variation. All adjustments to balance flexion gap were made by femoral component position adjustment. Most commonly this required external rotation of the femoral component. Once adjustments are made, gap laxity was again assessed and recorded. Only if the knee couldn’t be balanced within the boundaries set in Table 1, were soft tissue releases planned [4].

The traditional teaching of a balanced TKA, either through measured resection or gap balancing techniques, has been to achieve equal medial and lateral extension and flexion gaps. Previous studies would suggest that the native knee is not symmetrically balanced [5, 25]. More recently the need for a balanced lateral flexion gap has been questioned with several authors suggesting the natural variation in lateral flexion gap should be observed and replicated with a looser lateral flexion space to achieve improved kinematics [19, 21, 23]. This study assessed balance in extension (difference between medial and lateral gaps at 10° flexion), medial (difference between medial extension gap and medial flexion gap) and overall (the maximal imbalance observed, either extension or medial) and considered balance to be within 2 mm. Equal gaps were the preferential target.

Following virtual balancing all bone cuts were executed and trial implants inserted. Balance was assessed and final insert thickness determined by surgeon assessment of coronal and sagittal laxity. Patella was then resurfaced and definitive implants inserted. Final assessment of balance with quantitative measures of extension and flexion laxity made and recorded. Any requirement for soft tissue release for balance was also recorded.

### Statistical analysis

Statistical analysis was undertaken using IBM SPSS (IBM SPSS, V 27, Armonk, NY, IBM Corp) software package. Group percentages were calculated, and independent samples proportions tests were undertaken with two-sided Wald HO tests to assess for significance between groups. Student *t* tests were used to compare individual means and chi-square test for categorical data. The cut-off for statistical significance was defined as *p* < 0.05. Comparisons were made between the initial planned gaps for MA and KA plans, planned gaps after functional adjustment to balance the knee and measured gaps after final implantation. A priori power analysis was not possible due to the unknown incidence of overall balance with each alignment philosophy as measured by the Mako software. Post-hoc analysis confirmed adequate sample size to compare FA, MA and KA for incidence of overall balance.

## Results

130 TKAs planned with MA alignment and 170 TKAs planned with KA alignment were included in this study. Patient demographics, initial bony alignment, pre-operative Oxford Knee Scores (OKS) and pre-operative ROM are shown in Table 2, indicating no significant differences between the groups. The groups were analysed by CPAK classification with no difference seen in the distribution (NS).

**Table 2:** Patient group demographics and pre-operative native alignment

	MA plan	KA plan	*p*
Gender ratio (male:female)	65:65	74:96	NS
Mean age years	65.5	67.2	NS
Pre-operative mean BMI (kg/m^2^)	32.0	32.1	NS
Mean arithmetic hip knee ankle angle	− 0.9°	− 1.0°	NS
Mean medial proximal tibial angle	86.5	86.0	NS
Mean lateral distal femoral angle	87.0	87.0	NS
Pre-operative mean Oxford Knee Score	22.2	22.6	NS
Pre-operative mean range of motion-flexion	115.2°	115.5°	NS

**Fig. 1:**
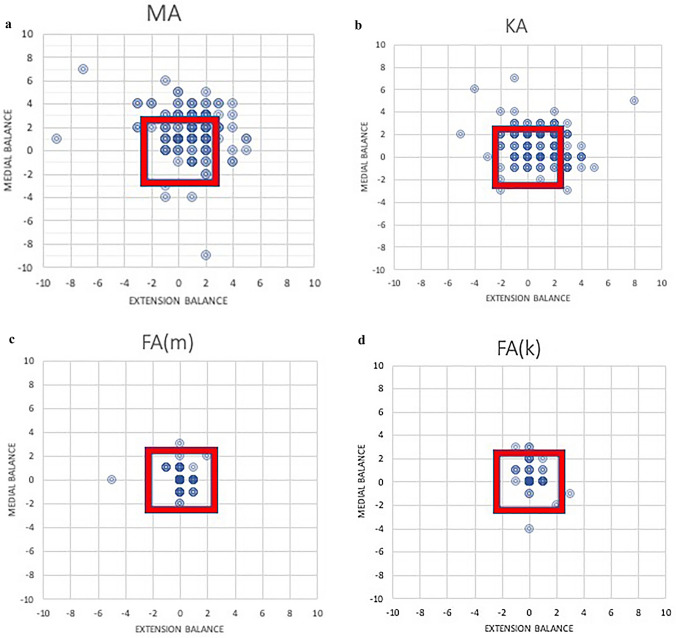
Scatter plot demonstrating the balance of knee with different alignment plans. The x-axis shows extension balance [difference between medial extension gap and lateral extension gap measured at 10° of flexion (mm)] and the y-axis shows medial balance [difference between medial extension gap and medial flexion gap (mm)]. The red box indicates accepted 2 mm of balance. **a** Mechanical alignment plan (MA). **b** Kinematic alignment plan (KA). **c** Functional alignment with mechanical starting point plan [FA(m)]. **d** Functional alignment with kinematic starting point plan [FA(k)]

Functional alignment obtained significantly better balance in extension, medially and overall than both MA and KA alignment (Table 3). Overall balance was observed in 97% of FA knees, 73% of KA knees and in 55% of MA knees. The difference between KA and MA was also significant (*p* = 0.002). The variation in balance between techniques is demonstrated in the scatter plots (Fig. 1).

**Table 3:** Number of knees balanced with initial surgical plan and subsequent functional alignment plan with percentage of cohort in brackets

	Balance achieved	MA 130 knees	FA(m) 130 knees	*p* value	KA 170 knees	FA(k) 170 knees	*p* value
Extension balance	≤ 1 mm	74 (57%)	128 (98%)	< 0.001	96 (56%)	168 (99%)	< 0.001
	≤ 2 mm	109 (84%)	129 (99%)	< 0.001	141 (83%)	169 (99%)	< 0.001
Medial balance	≤ 1 mm	58 (45%)	125 (96%)	< 0.001	91 (54%)	159 (94%)	< 0.001
	≤ 2 mm	86 (66%)	129 (99%)	< 0.001	149 (88%)	165 (97%)	< 0.001
Overall balance	≤ 1 mm	37 (29%)	124 (95%)	< 0.001	50 (29%)	158 (93%)	< 0.001
	≤ 2 mm	72 (55%)	128 (98%)	< 0.001	124 (73%)	164 (97%)	< 0.001

Mechanical axis alignment (MA). Functional alignment from a mechanical axis plan [FA(m)]. Kinematic axis alignment (KA). Functional alignment from a kinematic alignment plan [FA(k)]

The FA surgical plan (Table 4) showed significant differences in all alignment parameters except arithmetic hip knee ankle angle (aHKA) between FA(m) and FA(k). Having an individualised plan based on kinematic axis alignment resulted in the functionally aligned knees having an equivalent JLO to the KA plans (NS). There was no difference in balance obtained between the FA(m) and FA(k) cohorts (NS).

**Table 4:** Coronal alignment with functional adjustment of surgical plan

	FA(m)	FA(k)	*p* value
Femoral coronal alignment	0.0° (− 3,3)	2.5° (− 3,6)	< 0.001
Tibial coronal alignment	− 1.5° (− 6,3)	− 4.0° (− 6,3)	< 0.001
aHKA	− 1.5° (− 6,3)	− 1.5° (− 6,3)	NS
Joint line obliquity	178.5°	173.5°	< 0.001
Femoral rotation to SEA	2.0°	− 1.0°	< 0.001

Values are expressed as a mean in degrees with range in brackets. Positive numbers are valgus and negative numbers are varus. Femoral rotation: positive is externally rotated to the surgical epicondylar axis (SEA). Functional alignment from a mechanical axis plan [FA(m)]. Functional alignment from a kinematic alignment plan [FA(k)]

Functional alignment was seen to differ from both MA and KA with respect to alignment achieved. All differences were highly significant when comparing MA to FA (*p* < 0.001). When comparing FA to KA there was no difference in aHKA or MTPA. There were small but significant differences in LDFA (87.5° vs 87° *p* = 0.002) and JLO (173.5 vs 172.5, *p* = 0.008). Femoral rotation reached significance with FA having less internal rotation relative to the trans-epicondylar axis (1° vs 2°, *p* < 0.001).

Functional alignment resulted in a deviation of final limb alignment away from the native aHKA of greater than 1° in 24% of patients. In this subgroup the mean deviation was 2.5°.

This balance was maintained after prosthesis implantation with no significant difference in balance between groups (NS). Likewise, there was no difference in the balance seen with the virtual assessment and the final assessment once implantation of TKA occurred in either group (NS). There were two soft tissue releases performed for coronal balance in the FA(m) group and four performed in the FA(k) group. There were three lateral retinacular releases performed for patellofemoral tracking in the FA(m) group and four in the FA(k) group. The final balance achieved post-implantation of components was maintained with 99% of TKAs assessed as having adequate balance with a soft tissue release rate for tibiofemoral balance of 2% overall.

## Discussion

The most important findings of this were that FA provides a more balanced TKA than either MA or KA without the need for altering the soft tissue envelope of the knee. If it is accepted that more than 2 mm of imbalance would require soft tissue release to adequately balance the TKA, then FA would result in fewer soft tissue releases. Functional alignment, with pre-resection bony balancing, consistently achieved a balanced TKA with soft tissue releases for coronal balance required in 2% of TKAs.

The number of unbalanced TKA with KA would be less if the surgeon follows the technique described by Howell [12] and recuts the tibia to adjust extension balance. The imbalance between medial extension and flexion is more difficult to equalise with adjustments to tibial slope in addition to altering tibial coronal alignment, particularly if undertaken with conventional instrumentation.

Functional alignment with a KA initial plan, differs from KA in several ways. Firstly, following soft tissue assessment the depth of tibial cut can be altered to allow for bone preservation and the preferred polyethylene insert thickness to be chosen. Secondly, as balance is assessed prior to any bone cuts, alteration of femoral component rotation and sagittal position can be undertaken to equalise balance between flexion and extension, particularly on the medial side. Functional alignment places a greater emphasis on balancing the soft tissue envelope rather than remaining bound to equal resections of the femoral condyles. This emphasis is due to the possibility of bony remodelling throughout the arthritic process shifting the functional flexion axis.

The post-implantation balance achieved with FA utilising robotic assisted TKA (RATKA) was equivalent to that achieved virtually during pre-resection balancing. These findings further validate the work of Chang et al. [3] where balance was achieved in FA aligned TKAs. This is the first study to validate techniques with a pre-resection balance workflow achieving balance with either a KA or MA starting plan. Pre-resection balancing utilising this software is an accurate and reliable method for achieving soft tissue balance in TKA. The low soft tissue release rate required in this study supports the assessment of balance made.

Balance in TKA can be achieved by either soft tissue releases or alteration of component position or a combination of the two. With greater soft tissue release, there is a need for greater stability to be generated by the components as is seen in PCL sacrificing TKA. Complete release of the PCL increases the flexion gap by 4–5 mm[6]. When utilising cruciate retaining prostheses and particularly with the use of robotic assisted TKA, with a lower incidence of PCL injury [10], a tight medial flexion gap is more likely. Balancing a tight medial flexion gap in isolation with soft tissue release is technically demanding and often imprecise. This study demonstrated that using virtual balancing of the knee and intra-operative data on the soft tissue envelope allows the vast majority of TKAs to be balanced within the HKA boundaries of 6° varus and 3° valgus without soft tissue release. The ability to balance through precise adjustment of component position and the use of advanced technologies to accurately execute these planned cuts is likely to lead to greater reliability in achieving a balanced TKA. Improved balance has been shown to improve outcomes [9, 14].

Functional alignment was achieved with either a MA or KA starting plan and the resultant arithmetic HKA (aHKA) was equivalent. The most apparent difference between FA(m) and FA(k) is the component position with FA(k) resulting in increased tibial varus and femoral valgus. It is because of these differences that FA(k) is more likely to achieve all stated goals of FA as defined by Oessadik et al. [15], particularly with respect to restoring joint line plane and obliquity. This study demonstrated the mean joint line obliquity was significantly altered with MA planning.

This study has several limitations. This was a sequential cohort rather than randomised study. The groups were assessed as demographically, anatomically and clinically equivalent. The comparison between MA, KA and FA were using virtual measurements within the same software package rather than following completion of the surgical procedure. The gap measurements obtained were manual and could not have the force utilised quantified. The effect of this was minimised by having a single experienced surgeon using a consistent technique throughout the study. The KA technique did not allow for further adjustments of tibial position following assessment of gaps as per Howell’s technique. This would have improved the balance seen in the KA group but not addressed the imbalance on the medial side between extension and flexion. Balance measurements were virtual and prior to soft tissue releases that would be part of these techniques. Soft tissue releases can be undertaken with these techniques to achieve a balanced TKA.

## Conclusion

Functional alignment with pre-resection balancing results in improved balance compared to either mechanical or kinematic alignment in total knee arthroplasty prior to requiring soft tissue releases. The virtual balance obtained was replicated by the post-implantation values obtained validating the pre-resection balancing technique for robotic assisted TKA.

Functional alignment component position is significantly affected by the initial plan, with a kinematically aligned initial plan resulting in more oblique component position and joint line than a mechanically aligned plan. The clinical outcomes of functional alignment TKA need to be further assessed as does the optimal method for achieving the goals of the technique.
